# Effects of physical activity on internalizing problems in adolescents with autism spectrum disorder: the chain mediating effects of sport friendship quality and social-emotional competence

**DOI:** 10.3389/fpsyg.2025.1626831

**Published:** 2025-08-25

**Authors:** Yingbo Zhu, Xiao Li, Zhijuan Du

**Affiliations:** ^1^Physical Education College of Henan University, Kaifeng, China; ^2^Physical Education College of Linyi University, Linyi, China

**Keywords:** physical activity, autism spectrum disorder, internalizing problems, social-emotional competence, quality of sports friendships

## Abstract

**Background:**

Adolescents with autism spectrum disorder (ASD) often experience identity confusion, social difficulties, and internalizing symptoms such as anxiety and depression. Physical activity offers opportunities for peer interaction and teamwork, which may help alleviate negative emotions. This study aims to investigate the pathways through which physical activity influences internalizing problems in adolescents with ASD.

**Methods:**

A total of 436 adolescents with ASD were recruited using a combination of random and convenience sampling. Participants were assessed using the Physical Activity Rating Scale, the Strengths and Difficulties Questionnaire, the Social-emotional Competence Scale, and the Sport Friendship Quality Scale. Data were analyzed using SPSS 27.0 and Process 4.1 to examine the relationships among the four variables.

**Results:**

(1) Physical activity predicted a decrease in internalizing problems, and positively predicted sport friendship quality and social–emotional competence. (2) Both sport friendship quality and social–emotional competence independently mediated the relationship between physical activity and internalizing problems. (3) Additionally, a sequential (chain) mediation effect was identified, whereby physical activity influenced internalizing problems through both mediators in sequence.

**Conclusion:**

These findings suggest that physical activity holds substantial potential for mitigating internalizing symptoms among adolescents with ASD and supports the development of sport-based friendships and social-emotional skills. Future research should consider age-specific characteristics and individual preferences for activity types to identify the most effective interventions for enhancing friendship, social–emotional competence, and mental health in this population.

## 1 Introduction

Autism Spectrum Disorder (ASD) is a neurodevelopmental disorder characterized by significant social communication impairments and repetitive behaviors (Guha, 2014). The diagnosis of ASD is often accompanied by social stigma and is associated with poorer mental health outcomes during childhood and adolescence. Notably, comorbid internalizing disorders are prevalent in ASD, and these issues are often subtle and difficult to detect. Such problems can lead to social isolation, further exacerbating social functional impairments by reducing opportunities for social participation and interaction (Hilton et al., 2008; Lounds Taylor et al., 2017; Stewart et al., 2006).

Internalizing problems are typically manifested through anxiety, depression, and social withdrawal, which are directly inward symptoms (Reijntjes et al., 2010). The prevalence of internalizing and mental health issues in children and adolescents with ASD is significantly higher compared to typically developing (TD) children (Chandler et al., 2016). Anxiety and depression are the most common mental disorders among children and adolescents with ASD, and these disorders often co-occur or develop sequentially, showing high comorbidity rates, posing a substantial threat to both physical and mental health (Garber and Weersing, 2010; Merikangas et al., 2010). Anxiety symptoms in individuals with ASD tend to exacerbate core autism features, including social impairments, sensory abnormalities, and repetitive behaviors, and may be associated with the development of depression (Lai et al., 2019). Therefore, it is crucial to identify key factors that effectively alleviate internalizing problems behaviors in ASD adolescents when considering resource allocation and intervention strategies.

Intervention strategies for internalizing problems mainly include pharmacotherapy and psychological interventions (Aldridge et al., 2022). However, pharmacological treatments mainly used to manage co-occurring conditions associated with ASD can lead to side effects. In contrast, the use of psychotherapy and other non-pharmacological approaches remains limited, primarily due to high costs and constrained healthcare resources (Cipriani et al., 2016; Sharma et al., 2016). Therefore, in recent years, researchers have increasingly focused on modifiable lifestyle factors, particularly the role of physical activity (PA) in addressing adolescent internalizing problems (Kong et al., 2023; Zhang et al., 2022). Previous studies have shown that regular participation in PA has a positive impact on the mental health of TD individuals (Fedewa and Ahn, 2011). For example, PA has been significantly associated with reduced symptoms of anxiety and depression, as well as improved self-concept, attention, memory, and academic performance (Bremer et al., 2016). Physically inactivity may negatively impact social and psychological development, leading to social difficulties (such as social isolation and reduced social interaction), which in turn limits the formation of coping strategies and makes individuals more likely to avoid PA (Cairney et al., 2013; Hill et al., 2024).

Adolescents with ASD face greater social barriers and are less physically active than their TD peers and other disability groups (Bandini et al., 2013; Mitchell et al., 2010). As a result, individuals with ASD typically face increased prevalence, high prevalence of obesity and overweight, and health-related low levels of physical fitness (Stanish et al., 2017). However, most of the existing research on the improvement of PA on internalizing problems has focused on TD individuals, which makes the current relationship between PA and internalizing problems in adolescents with ASD unclear; therefore, the aim of this paper is to explore the relationship between PA, internalizing problems, quality of sports friendships, and social–emotional competence (SEC) in adolescents with ASD.

### 1.1 Physical activity and internalized behavior

PA plays a crucial role in the intervention of negative emotions, primarily through physiological and psychological mechanisms. In terms of the physiological mechanism, the endorphin hypothesis suggests that exercise promotes the secretion of endorphins by the pituitary gland, and endorphins are closely related to the improvement of positive emotions (Ai et al., 2012; Li et al., 2017). Considering the sensitive and critical periods in the growth and development of adolescents, aerobic exercise not only stimulates the release of endorphins and activates the neuroendocrine system to alleviate pain and improve mood, but also increases hippocampal volume and promotes the secretion of brain-derived neurotrophic factor (BDNF), thus effectively alleviating symptoms of anxiety (Erickson et al., 2011). Ishii et al. (2016) found that regular PA is positively related to children’s self-efficacy and negatively related to their emotional and behavioral problems. In the laboratory, adolescents’ PA was shown to be positively associated with mood dimensions, that is, energetic arousal and valence and negatively associated with calmness (Shahane et al., 2024).

Given the effectiveness of PA in improving the emotional and behavioral functioning of TD children, adolescents with ASD may also benefit from it. A few studies have indicated that PA can help improve the social functioning of children with ASD. Rosenthal-Malek and Mitchell (1997) found that a 10-session, 20-min jogging intervention significantly reduced self-stimulatory behaviors in children with ASD. Additionally, two systematic reviews and one meta-analysis have investigated the effects of PA on behavioral functioning in children with ASD. The results of the meta-analysis indicate that PA leads to an overall effect size of 37.5% in improving sports skill, social skills, and other related areas (Bremer et al., 2016; Petrus et al., 2008; Sowa and Meulenbroek, 2012). These studies consistently suggest that PA may be an effective intervention for improving social behavioral functions in adolescents with ASD. Notably, based on a three-wave longitudinal dataset from the Korean Children and Youth Panel Survey (KCYPS), which followed 2,092 middle school students over a three-year period, the study found that PA measured at Time 1 significantly predicted lower internalizing problems at Time 2 for both boys (*β* = −0.16, *p* < 0.05) and girls (*β* = −0.11, *p* < 0.05) (You et al., 2021). However, previous studies have paid less attention to the relationship between PA and internalizing problems in adolescents with ASD. Additionally, the validation of possible psychologically mediated variables between the two remains pending. Therefore, the present study aimed to build on previous research and continue to explore in depth the relationship between PA and internalizing problems in adolescents with ASD.

### 1.2 Mediating role of sport friendship quality

Compared to TD peers, adolescents with ASD often experience more social–emotional difficulties and lower friendship quality (Ghaziuddin et al., 2002). The importance of peer relationships in the development of children and adolescents cannot be overstated. Recognition and support from a broad peer group (such as classmates and teammates) significantly influence an individual’s sense of self-worth and emotional states, including both positive and negative emotions (Weiss and Smith, 2002). PA is considered an ideal environment for promoting positive social interactions in children with ASD (Zhao and Chen, 2018). For instance, studies on TD adolescents have shown that participation in extracurricular activities has a positive impact on social adaptation and emotional regulation, particularly in reducing symptoms of depression and alleviating feelings of loneliness (Fredricks and Eccles, 2005). Similarly, research on adolescents with developmental disorders has found that participation in school activities not only increases social interactions but also fosters collaboration with non-disabled peers (Rynders et al., 1993). Therefore, ASD adolescents may find it easier to form peer relationships through PA, thereby enhancing friendship quality.

The quality of sports friendships refers to the close relationships that adolescents develop through interactions in sports contexts. In these interactions, they gain trust and intimacy, receive comfort, support, and help from friends, and obtain acceptance from their peer group through these connections (Han et al., 2013). For children who are typically excluded or bullied by their peers, friendship acts as a buffer, reducing the occurrence of depressive symptoms (Bukowski et al., 2010). The presence of friends and the quality of friendships are crucial for preventing psychological symptoms, with certain friendship characteristics, such as security, companionship, and helpfulness, being associated with fewer mental health issues (Wood et al., 2017). In contrast, friendships with conflict are linked to more internalizing symptoms (La Greca and Harrison, 2005). Bosacki et al. (2007) found study hypothesizes that, compared to those with lower-quality friendships or no close relationships, adolescents who maintain close friendships may be less likely to experience internalizing problems. However, there is relatively little research on the relationship between sport friendship quality and adolescent mental health. Therefore, this study hypothesizes that the quality of sports friendships mediates the relationship between PA and internalizing problems in adolescents with ASD.

### 1.3 Mediating effects of social-emotional competence

SEC refers to an individual’s ability to understand and regulate emotions in complex social situations, as well as to establish and maintain positive social relationships (Martzog et al., 2016). The inward and social–emotional functioning model proposed by Coplan et al. (2020) suggests that children’s social communication skills and emotional regulation abilities play a protective role in preventing internalizing problems. Research has also shown that individuals with high SEC are more likely to employ adaptive emotional regulation strategies, thereby reducing the impact of negative emotions on their mental health (Mikolajczak et al., 2008). Therefore, children with high SEC are better able to regulate negative emotions, reduce social avoidance, lower levels of anxiety and stress, and provide positive support for mental health development.

Malinauskas and Malinauskiene (2021) research suggests that physical education can enhance adolescents’ SEC in areas such as emotional understanding, empathy, cooperation, self-control, and social skills, thereby validating the positive role of PA in developing SEC. Social control theory posits that PA plays a positive role in imparting social norms, fostering positive interpersonal relationships, enhancing mutual understanding, and alleviating psychological stress (Li, 1992). Furthermore, a longitudinal cohort study involving 2,278 students aged 10 to 14 found that a higher frequency of participation in PA was associated with increased perceived social competence over time (*b* = 0.06, 95% CI [0.04, 0.08]). This predictive relationship remained consistent across gender and types of activity (both school-based and out-of-school), supporting the notion that PA contributes to the development of socio-emotional competence (Bedard et al., 2020). These studies suggest that PA not only helps maintain behavioral norms but also improves individuals’ ability to manage relationships with others and the community. A meta-analysis showed that the development of SEC significantly contributes to social behavior (*ES* = 0.28), alleviation of problematic behavior (*ES* = 0.24), and emotional distress (*ES* = 0.28) (Durlak et al., 2011). In summary, PA may indirectly influence internalizing problems in adolescents with ASD by enhancing their SEC.

### 1.4 Chain mediation of sport friendship quality and social-emotional competence

The quality of sports friendships may be significantly related to SEC. Specifically, interactions among peers provide a conducive learning environment for the development of SEC (Yang et al., 2017). Through engaging in peer interactions, individuals are able to develop and apply social–emotional skills, and those who are accepted by their peers tend to perform better in emotional understanding, expression, and adoption (Ma et al., 2011). In contrast, students who experience peer rejection are excluded from social activities, leading to more negative experiences. These individuals tend to have poorer emotional regulation abilities and are more likely to exhibit problematic behaviors, which hinders the development of SEC. As Bauminger et al. (2008) hypothesized, individuals with ASD who have reciprocal friendships perform better in social skills and SEC than those without friends.

In educational settings, body-oriented experimental interventions have been studied with preschoolers and have shown positive effects of PA on their peer acceptance, SEC, and emotion regulation strategies (Dias Rodrigues et al., 2022). Further studies with normally developing children have carefully delineated PA interventions and found that PA combined with play had more significant effects on social interactions, SEC, and internalizing problems compared to psychomotor and dance. (Dias Rodrigues et al., 2022). In studies with children and adolescents with ASD, a small number of studies have reported that PA interventions may be effective in improving their communication skills, social functioning, and quality of life, and that the earlier the intervention is initiated, the more significant the effects are (Chan et al., 2021). Although existing studies have not directly examined the relationship between motor friendship quality and SEC in PA and problem behaviors in ASD, they provide important theoretical support for the present study. In summary, motor friendship quality and SEC may play a chain-mediated role in the influence of PA on internalizing behaviors in adolescents with ASD.

### 1.5 Hypotheses of this study

**Figure 1:**
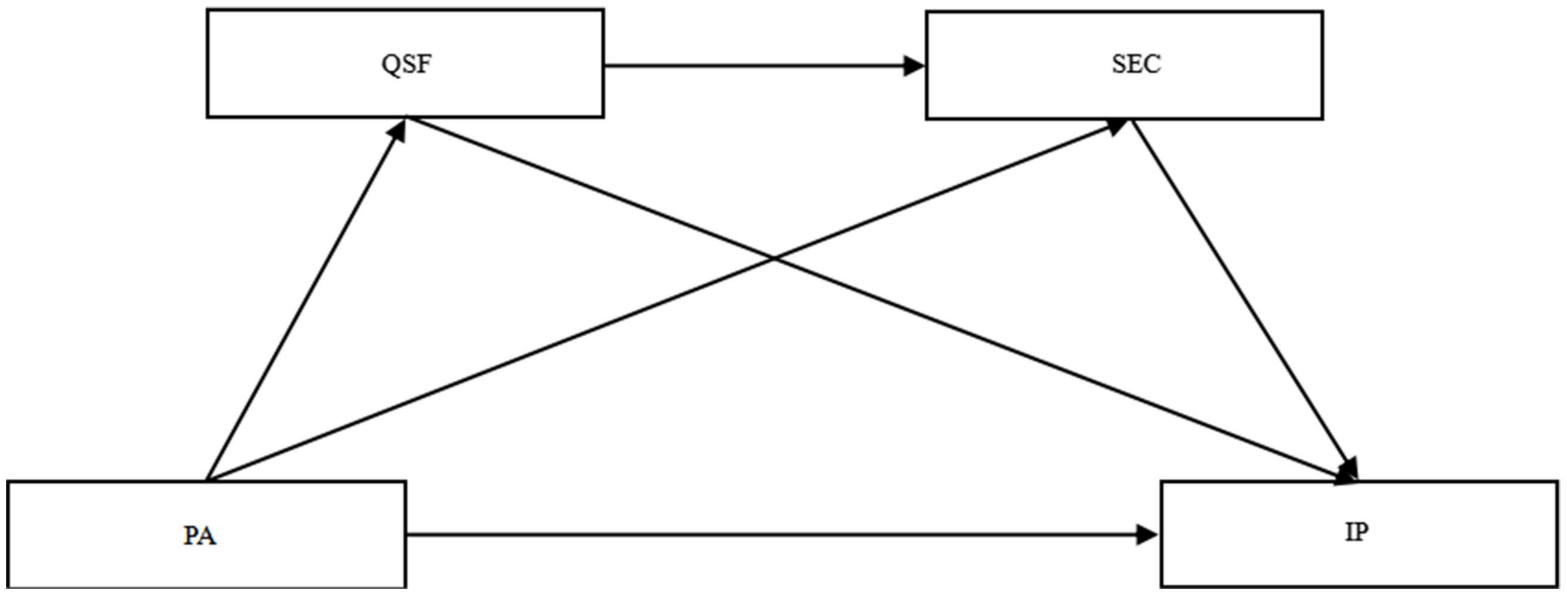
The theoretical model diagram. Physical activity = PA; Quality of sports friendship = QSF; Social–emotional competence = SEC; Internalizing problems = IP.

This study aims to examine the chain mediating roles of sports friendship quality and SEC in the relationship between PA and internalizing problems among adolescents with ASD, in order to deepen the understanding of how PA influences adolescent mental health through psychosocial mechanisms. Previous studies have shown that PA, sports friendship quality, and SEC are each closely associated with internalizing problems such as depression and anxiety in adolescents. However, empirical research on how these factors interact specifically in adolescents with ASD remains limited and inconclusive. Accordingly, the present study seeks to clarify the relationship between PA and internalizing problems in adolescents with ASD, with a particular focus on the potential chain mediating pathways involving sports friendship quality and SEC. The findings of this study are expected to provide a theoretical basis for the prevention and intervention of internalizing problems in this population. Based on existing literature, the following four hypotheses are proposed (see Figure 1):

*H1*: PA predicts a decrease in internalizing problems in adolescents with ASD.

*H2*: The quality of sports friendships plays an independent mediating role between PA and internalizing problems in adolescents with ASD.

*H3*: SEC plays an independent mediating role between PA and internalizing problems in adolescents with ASD.

*H4*: The quality of sports friendships and SEC play a chain mediating role between PA and internalizing problems in adolescents with ASD.

## 2 Methods

### 2.1 Sample

**Figure 2:**
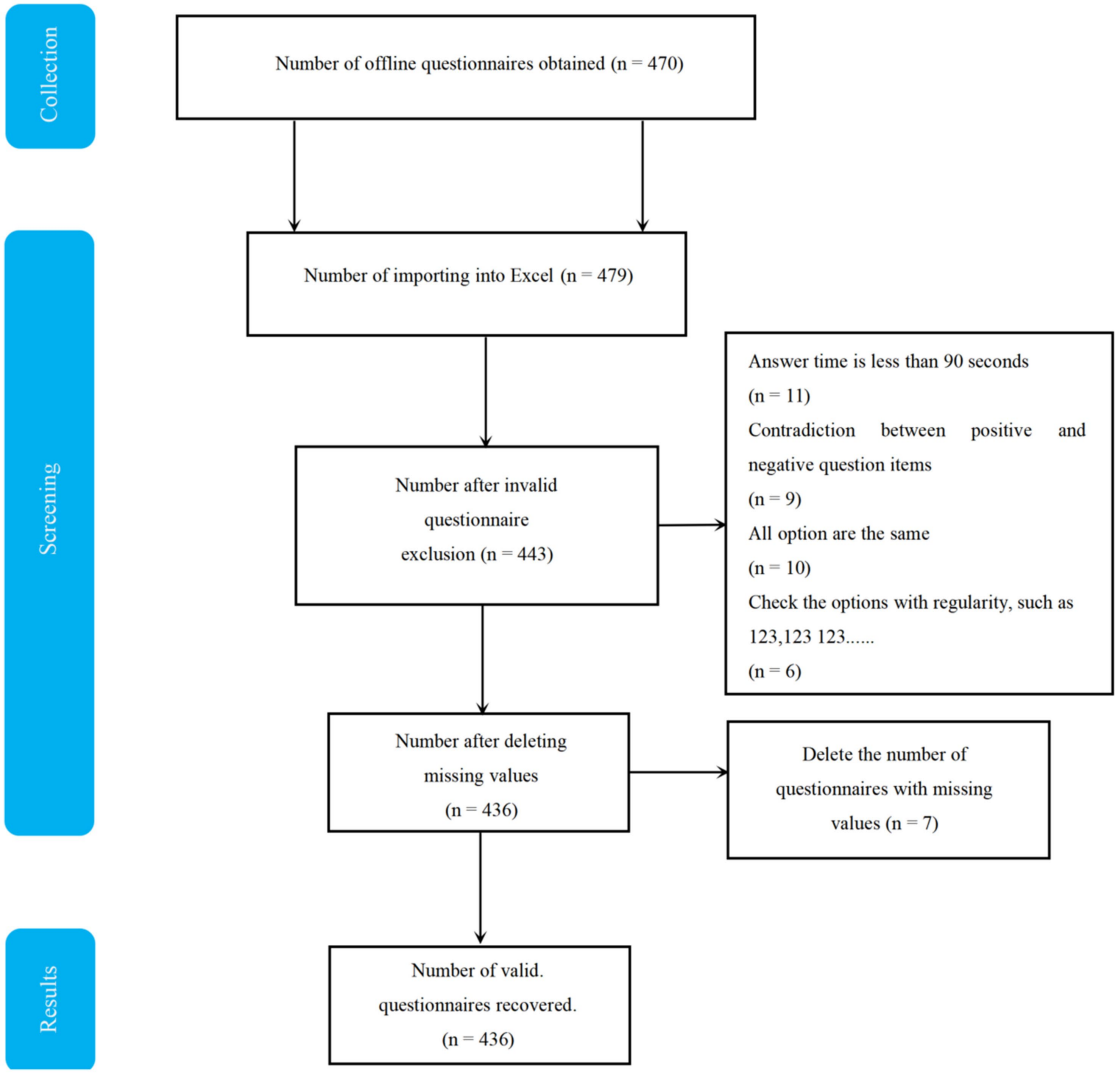
Steps in the screening process for research samples.

A total of 463 adolescents with ASD participated in this study. They were recruited from local branch clinics and special education institutions. The inclusion criteria were as follows: (1) ASD diagnosis made by pediatric psychologists and experienced developmental-behavioral pediatricians at local hospitals based on the criteria in the fifth edition of the Diagnostic and Statistical Manual of Mental Disorders (DSM-5) (Regier et al., 2013); (2) the ability to follow instructions with assistance from the researchers; (3) no history of reading disorders. The exclusion criteria were: (1) other medical conditions that restrict PA (e.g., asthma, epilepsy, heart disease); (2) adolescent with severe physical illnesses or sensory impairments (e.g., blindness or deafness); (3) adolescent with other independent neurodevelopmental disorders or neurological conditions; (4) adolescent with acute or chronic diseases. A total of 479 questionnaires were distributed, with 436 valid responses received, resulting in a response rate of 91.0%. The specific screening process is shown in Figure 2. Of the 436 valid questionnaires collected, 254 (58.25%) were predominantly male and 182 (41.74%) were female; the average age of adolescents was (12.37 ± 2.23). Detailed demographic characteristics are presented in Table 1.

**Table 1:** Distribution of basic information on adolescents (*N* = 436).

Demographic variables		Number	Proportion %
Age	12.37 ± 2.23	436	100%
Gender	Male	254	58.25%
	Female	182	41.74%
Grade level	Sixth grade	86	19.72%
	First year	132	30.27%
	Second year	133	30.50%
	Third year	85	19.50%
Place of birth	Rural	175	40.13%
	Urban	261	59.86%
Only child or not	Yes	88	20.18%
	No	348	79.81%
Boarding or not	Yes	305	69.95%
	No	131	30.04%
Behavioral intervention or not	Yes	327	75.00%
	No	109	25.00%
Year of diagnosis	0–3 years	371	85.10%
	More than 3 years	65	14.90%

### 2.2 Procedure

The study was conducted from March to April 2025 in Henan, China, using a combination of purposive and convenience sampling methods. The present study was approved by the Biomedical Research Ethics Subcommittee of Henan University, and written informed consent was obtained from both the adolescents and their parents. The researchers conducted one-on-one surveys with participants using paper-based questionnaires. All diagnostic and screening procedures were completed by physicians from different schools and trained research assistants prior to the commencement of the study. The questionnaires were completed by the participants themselves and collected on-site. After collection, the researchers carefully checked the completeness of the responses and immediately sought clarification from the participants if any issues were identified. All questionnaires were administered and retrieved in person.

### 2.3 Measurement

#### 2.3.1 Physical activity

In this study, the Physical Activity Rating Scale (PARS-3), revised by Liang (1994), was used to assess the PA levels of adolescents with ASD. This scale has been widely applied in related research (Wu et al., 2024) and evaluates three dimensions of PA: intensity, duration, and frequency. Each item is rated on a five-point Likert scale. A sample item is: “How often do you engage in the above physical activities?” The total PA score is calculated using the formula: PA amount = Intensity × Duration × Frequency. Both intensity and frequency are scored from 1 to 5, while duration is graded from 1 to 5 and scored from 0 to 4. The total score ranges from 0 to 100, with higher scores indicating greater levels of PA. Based on the scoring criteria, PA levels are categorized as follows: ≤ 19 = low activity; 20–42 = moderate activity; ≥ 43 = high activity. In the present study, the Cronbach’s *α* coefficient of the scale was 0.74, indicating acceptable internal consistency reliability.

#### 2.3.2 Internalizing problem behavior measures

Children’s internalizing problem scores were assessed using the self-report version of the Strengths and Difficulties Questionnaire (SDQ) (Goodman, 1997). The SDQ is a globally recognized screening tool designed to prompt students to report on their behaviors and emotions over the past 6 months (Goodman et al., 1998). Based on previous research, internalizing scores were derived from the SDQ’s Emotional Symptoms subscale (5 items) and Peer Problems subscale (5 items). The Chinese version of the SDQ has been validated among adolescents in China, demonstrating good reliability and validity (Zhang et al., 2019), and has been widely used in the assessment of internalizing problems (Bekkhus et al., 2023; Zhou et al., 2024). The scale adopts a three-point response format (0 = not true, 1 = somewhat true, 2 = certainly true). The total score for all items was calculated, with higher scores indicating a higher level of internalizing problem behaviors. In this study, the Cronbach’s *α* for the scale was 0.88, indicating high internal consistency.

#### 2.3.3 Sports friendship quality

This study used the Youth Sports Friendship Quality Scale developed by Weiss and Smith (1999) and revised by Zhu et al. (2010) to adapt it to the Chinese cultural context. The scale includes 6 dimensions and 25 items. All items were scored using a 5-point Likert scale, where 1 represents “Strongly Disagree” and 5 represents “Strongly Agree.” A higher total score indicates better quality of sports friendships. Previous studies have shown that the scale has good applicability within adolescent populations (Ji, 2021). In this study, the Cronbach’s α for the scale was 0.94, indicating high internal consistency.

#### 2.3.4 Social–emotional competence

This study adopted the framework established by the Collaborative for Academic, Social, and Emotional Learning (CASEL) (Wu et al., 2024), which divides SEC into five dimensions. The scale consists of 27 items, with response options ranging from “Strongly Disagree” to “Strongly Agree” scored from 1 to 5. In this study, the Cronbach’s α for the scale was 0.93, indicating high internal consistency.

### 2.4 Data analysis methods

This study utilizes SPSS 27.0 for data processing. Harman’s single-factor method was employed to test the common method bias of the data. Descriptive statistical analysis was performed on the variables to understand their score distribution. Pearson correlation analysis was used to examine the relationships between variables. Hypothesis testing of the chain mediating model was carried out by constructing a multiple linear regression model to analyze the main effects, and further testing of chain mediating effect was examined using Model 6 in the SPSS macro plugin Process 4.1 developed by Hayes (2012). The mediation effect was analyzed using the Bootstrap method with a 95% confidence interval and 5,000 samples for effect testing.

## 3 Results

### 3.1 Common method bias test

Because all variables were assessed through self-report, Harman’s single-factor test was used to assess possible common method bias (Podsakoff et al., 2003). The results show that 13 common factors with initial eigenvalues greater than 1 were extracted using principal component analysis, which accounts for 67.902% of the total variance. The unrotated cumulative variance percentage for the first factor is 28.142%, well below the 40% threshold, suggesting that the data in this study does not suffer from severe common method bias.

### 3.2 Descriptive statistics and correlation analysis

Table 2 lists and describes the means, standard errors and correlations for PA, internalizing problems, quality of sports friendship and SEC. PA levels were categorized as low (≤ 19 points), moderate (20–42 points), or high (≥ 43 points). Among the participants, 54.8% of adolescents with ASD were classified as having low activity levels, 35.1% as moderate, and only 10.1% as high. This indicates that adolescents with ASD lack PA and have lower PA competence. The score for internalizing problem behaviors (13.46 ± 4.52) suggests that the internalizing problem behaviors of adolescents with ASD are at a moderate level. The score for quality of sports friendships (82.60 ± 20.99) indicates a good degree of recognition of sports friendship (above the moderate level by 62.5). The score for SEC (89.52 ± 21.16), indicates that adolescents with ASD have a good recognition of their social–emotional abilities (above the moderate level by 67.5).

**Table 2:** Means, standard deviations and correlations among all variables (*N* = 436).

Variable	*M*	*SD*	1	2	3	4
1. Internalizing problems	13.46	4.52	1	–	–	–
2. Physical activity	22.03	18.52	−0.42**	1	–	–
3. Quality of sports friendship	82.60	20.99	−0.46**	0.43**	1	–
4. Social–emotional competence	89.52	21.16	−0.44**	0.44**	0.57**	1

*p < 0.05, **p < 0.01.

The results of the correlation analysis of variables show that the independent variable, PA, has a significant negative correlation with the dependent variable, internalizing problem behaviors (*r* = −0.416, *p* < 0.01). The mediating variables, quality of sports friendships. (*r* = −0.459, *p* < 0.01) and SEC (*r* = −0.443, *p* < 0.01), also have significant negative correlations with internalizing problem behaviors. Furthermore, PA is positively correlated with quality of sports friendships (*r* = 0.425, *p* < 0.01) and SEC (*r* = 0.441, *p* < 0.01) to varying degrees. There is also a positive correlation between the mediating variables, quality of sports friendships and SEC (*r* = 0.572, *p* < 0.01) (see Table 2).

### 3.3 Regression analysis

To test whether PA, quality of sports friendships, and SEC could predict internalizing problems, we conducted hierarchical multiple regression analyses, the results of which are shown in Table 3. Prior to that, we conducted a diagnostic analysis of multicollinearity for the predictor variables, and the results showed that the variance inflation factor (VIF) values of the respective variables ranged from 1.31 to 1.60, which were all less than 10, indicating that the data in this study did not have any obvious multicollinearity problems and were suitable for further regression analysis and mediation effect tests.

**Table 3:** Results of regression analysis.

Variant		Dependent variable: IP								Dependent variable: QSF		Dependent variable: SEC	
		Model 1		Model 2		Model 3		Model 4		Model 5		Model 6	
		β	*t*	β	*t*	β	*t*	β	*t*	β	*t*	β	*t*
Constant		13.11	4.40	14.64	5.38	19.33	7.49	22.48	8.54	57.89	4.52	68.11	5.90
Control variable	Gender	0.74	1.69	0.65	1.64	0.63	1.71	0.65	1.81	−0.27	−0.14	0.54	0.33
	Age	0.36	1.03	0.23	0.72	0.43	1.47	0.31	1.05	2.54	1.71	−2.76	−2.10
	Grade level	−0.79	−1.04	−0.54	−0.78	−0.93	−1.44	−0.65	−1.03	−4.75	−1.46	5.95	2.07
	Place of birth	−0.58	−1.34	−0.02	−0.05	0.00	−0.01	0.11	0.31	0.19	0.10	2.53	1.52
	Only child or not	−1.75**	−3.15	−1.46***	−2.87	−1.79***	−3.79	−1.98***	−4.26	−4.08	−1.71	−4.13	−1.96
	Boarding or not	0.72	1.49	0.98	2.22	1.09*	2.66	1.03*	2.57	1.35	0.65	−1.29	−0.71
Independent variable	PA	–	–	−0.10***	−9.34	−0.06***	−5.46	−0.05***	−4.27	0.49***	9.82	0.27***	5.52
Mediating variable	QSF	–	–	–	–	−0.08***	−8.49	−0.06***	−5.57	–	–	0.47***	11.13
	SEC	–	–	–	–	–	–	−0.05***	−4.35	–	–	–	–
Model Fit	*R*	0.24		0.47		0.57		0.60		0.44		0.62	
	*R^2^*	0.06		0.22		0.33		0.36		0.20		0.39	
	Adjusted *R^2^*	0.04		0.33		0.32		0.35		0.18		0.38	
	*△R^2^*	0.06		0.22		0.33		0.36		0.20		0.39	
	*F*	4.31***		16.88***		26.24***		26.42***		14.94***		33.88***	

Physical activity = PA; Quality of sports friendship = QSF; Social–emotional competence = SEC; Internalizing problems = IP. *p < 0.05, **p < 0.01, ***p < 0.001.

First, in Model 1, we included demographic variables such as gender, age, grade level, place of birth, only child or not and boarding or not as control variables. Next, PA was entered into Model 2, and the results showed that it significantly predicted a decrease in internalizing problems (*β* = −0.10, *p* < 0.001). In addition, the results of Model 3 showed that the quality of sports friendships significantly predicted a decrease in internalizing problems (*β* = −0.08, *p* < 0.001). In Model 4, we further found that SEC also significantly predicted a decrease in internalizing problems (*β* = −0.05, *p* < 0.001).

In addition, as shown in Model 5, PA had a significant positive effect on quality of sports friendships (*β* = 0.49, *p* < 0.001). Finally, the results of Model 6 indicated that PA had a significant positive effect on SEC (*β* = 0.27, *p* < 0.001) and that quality of sports friendships also had a significant positive effect on SEC (*β* = 0.47, *p* < 0.001). This hierarchical regression analysis clearly demonstrated the interrelationships between PA, quality of sports friendships, and SEC, and each variable showed a significant effect in predicting internalization problems.

### 3.4 Mediating role test

**Figure 3:**
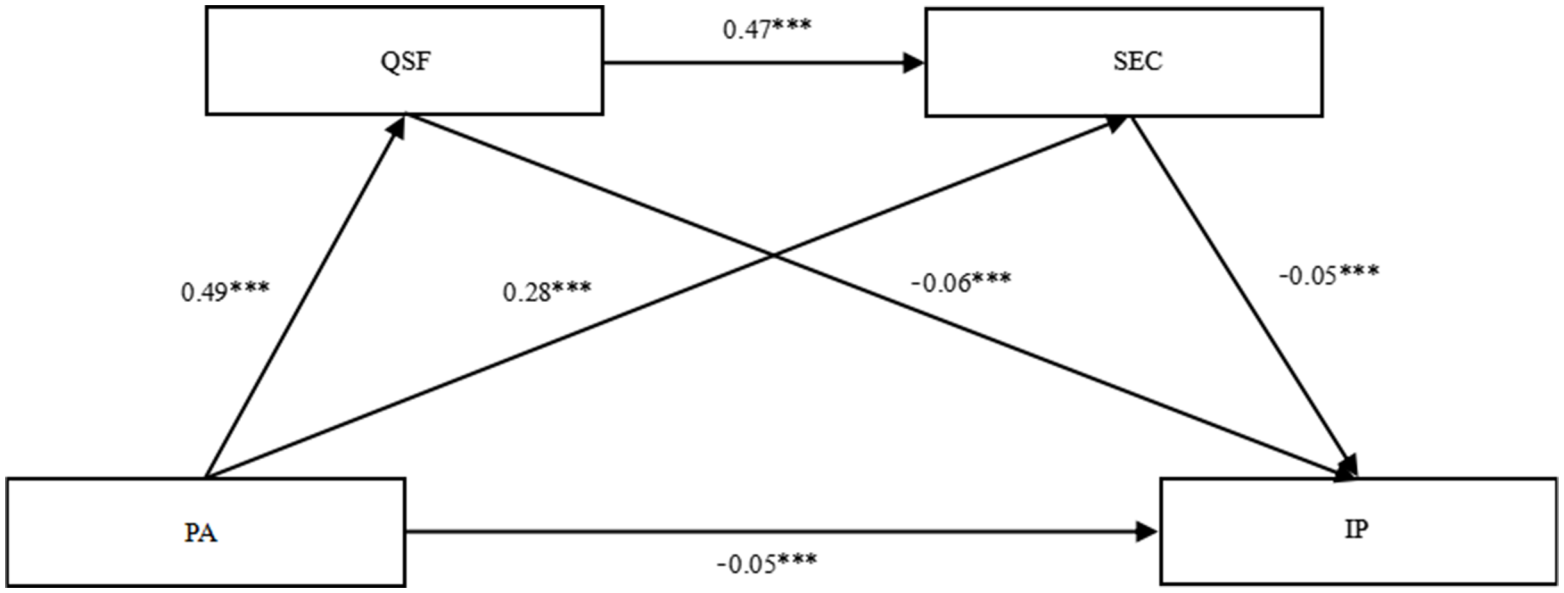
Chain mediation effects of quality of sports friendship and social-emotional competence in the relationship between physical activity and internalizing problems. Non-standardized path coefficient; Physical activity = PA; Quality of sports friendship = QSF; Social-emotional competence = SEC; Internalizing problems = IP.

The results of the chained mediation analyses (see Table 4; Figure 3) showed that PA had a significant direct effect on internalizing problems (*b* = −0.05, 95% CIs [−0.07, −0.03]). This direct effect accounted for 47% of the total effect. There was a significant indirect effect of quality of sports friendships and SEC between PA and internalizing problems (*b* = −0.05, 95% CIs [−0.07, −0.04]), and this indirect effect accounted for 53% of the total effect. These findings support the existence of a chain-mediated pathway whereby the quality of sports friendships and SEC jointly mediate the effect of PA on internalizing behaviors in adolescents with ASD, thus providing empirical support for Hypothesis 4.

**Table 4:** Mediating effects and effect sizes.

Path	Effect	*SE*	Bootstrap 95%CI		Percentage of total effect
			Lower	Upper	
Total effect	−0.10	0.01	−0.12	−0.08	–
Direct Effect	−0.05	0.01	−0.07	−0.03	47%
Total indirect effects	−0.05	0.01	−0.07	−0.04	53%
Ind1: PA → QSM → IP	−0.03	0.01	−0.04	−0.02	54%
Ind2: PA → SEC → IP	−0.01	0.00	−0.02	−0.01	25%
Ind3: PA → QSM → SEC → IP	−0.01	0.00	−0.02	−0.01	20%

In the path-specific mediation results for Ind1, the quality of sports friendships significantly mediated the effect of PA on internalizing behaviors in adolescents with ASD (*b* = −0.03, 95% CIs [−0.04, −0.02]), accounting for 54% of the total indirect effect. This finding supports Hypothesis 3, indicating that the quality of sports friendships serves as a mediator in the relationship between PA and internalizing behaviors in adolescents with ASD. The indirect effect Ind2 shows that SEC significantly mediates the impact of PA on internalizing behaviors in adolescents with ASD (*b* = −0.01, 95% CIs [−0.02, −0.01]), accounting for 25% of the total indirect effect. This finding supports Hypothesis H2, suggesting that SEC plays a mediating role in the effect of PA on internalizing behaviors in adolescents with ASD. The indirect effect Ind3: The joint mediating role of sports friendship quality and SEC in the impact of PA on internalizing behaviors in adolescents with ASD is significant (*b* = −0.01, 95% CIs [−0.02, −0.01]), accounting for 20% of the total indirect effect.

## 4 Discussion

The current study examines the relationships between PA, the quality of sports friendships, SEC, and internalizing problems, with a particular focus on the impact of PA on internalizing problems in adolescents with ASD and the mediating roles of sports friendship quality and SEC. The results indicate that PA, sports friendship quality, and SEC predict a decrease in internalizing problems among adolescents with ASD. Sports friendship quality and SEC each mediate, and together form a serial mediation, the effect of PA on internalizing problems in adolescents with ASD.

### 4.1 The effect of physical activity on internalizing problems

The results of this study suggest that PA predicts a reduction in internalizing problems among adolescents, supporting the neurobiological hypothesis that PA mitigates mental symptoms by altering brain structure or function (Stillman et al., 2020). In adolescents with ASD, higher levels of PA were significantly associated with fewer internalizing symptoms, consistent with Hypothesis 1. This finding corroborates prior research indicating that adolescents engaging in exercise reported fewer psychological and emotional disorders (He et al., 2018; Rodriguez-Ayllon et al., 2023).

This study reinforces the beneficial effect of PA on reducing internalizing problems in adolescents with ASD, consistent with previous findings. Even after controlling for individual and family-level covariates, PA remains significantly associated with decreased depressive symptoms in both TD adolescents and those with ASD (Eime et al., 2013; Panza et al., 2020). The effect is particularly evident when PA involves structured social interaction (Bohnert et al., 2019). Given adolescents’ developmental needs for identity formation and social acceptance, PA can support these processes by (1) fostering self-concept through goal achievement and perceived competence, and (2) enhancing social connectedness via peer support and group participation (Bohnert et al., 2019; Cruwys et al., 2013; Eime et al., 2013). Integrating prior evidence with the present findings, PA appears especially effective in alleviating internalizing symptoms among adolescents with mental health vulnerabilities, compared to general populations (Larun et al., 2006).

To promote PA among adolescents with ASD, schools are encouraged to implement diverse, structured, and group-based programs (e.g., football clubs, swimming courses). Programs should emphasize sports friendships and social–emotional skill development, creating inclusive environments that support both physical engagement and psychological well-being.

### 4.2 Mediating effects of quality of sports friendships between physical activity and internalizing problems

This study identified the quality of sports-based friendships as a partial mediator in the relationship between PA and internalizing problems, supporting Hypothesis 2. This result aligns with findings from TD adolescents (Bohnert et al., 2019). Among adults with ASD, close friendships have been linked to reduced loneliness, lower levels of depression and anxiety, and improved well-being (Mazurek, 2014). However, the contextual factors that facilitate friendship formation in adolescents with ASD remain insufficiently understood. While the cross-sectional design limits causal inference, PA may provide an enabling environment in which adolescents with ASD can acquire social skills and build meaningful peer relationships through shared goals and cooperative activities. These friendships may generalize to other social settings and contribute to broader social development. Notably, adolescents with ASD who did not participate in sports were twice as likely to experience difficulties initiating friendships and 2.35 times more likely to struggle in maintaining them, compared to their physically active peers (Pappagianopoulos et al., 2024).

According to the social-psychological mechanism theory, PA fulfills basic psychological needs—such as interpersonal connection—which may help mitigate mental health symptoms (Conley et al., 2020). Within PA settings, sports friendships represent a distinct form of peer relationship that enhances exercise engagement, alleviates loneliness, and fosters social competence in adolescents (Li, 2025). Consistent with prior research, our study found that higher-quality sports friendships were associated with fewer internalizing problems. This aligns with evidence suggesting that friendship is a key determinant of life quality in children with psychosocial challenges (Sigstad, 2016). Given that adolescence is a critical period for identity formation and social autonomy, friendship quality emerges as a salient indicator of mental health. Enhancing sports friendship quality may therefore serve as an effective intervention strategy for reducing internalizing symptoms in adolescents with ASD.

### 4.3 Mediating effects of social–emotional competence between physical activity and internalizing problems

This study confirms that SEC serves as a significant mediator between PA and internalizing problems, supporting Hypothesis 3 and aligning with previous findings. The physiological hypothesis suggests that exercise enhances emotional well-being by increasing neurochemicals such as norepinephrine, serotonin, and endorphins, which improve mood and induce euphoria (MacMahon, 1990). Individuals who engage in regular PA typically demonstrate stronger emotional regulation, social skills, and overall SEC (Harber and Sutton, 1984). Systematic reviews further indicate that PA-based interventions positively influence multiple dimensions of children’s SEC, especially in areas such as emotion recognition, empathy, and regulation strategies (Dias Rodrigues et al., 2022). In addition, PA provides opportunities for adolescents to develop core SEC skills, including sharing, cooperation, and empathy, thereby fostering holistic social–emotional growth (Wang et al., 2024).

Our findings further underscore the predictive role of SEC in reducing internalizing problems among adolescents with ASD, suggesting that SEC may function as a protective factor against emotional distress in this population. Prior research has highlighted the critical role of SEC in promoting healthy development among adolescents at risk for emotional and behavioral problems, as it buffers the adverse effects of environmental stressors (Lubans et al., 2012). This perspective is supported by the integrative model of emotional competence, social competence, and psychopathology proposed by Perren and Malti (2008), which posits that emotional competence not only influences mental health directly but also indirectly via social competence. This theoretical framework aligns with our findings: adolescents with higher SEC are better equipped to regulate their emotions, interpret others’ emotional cues, and adapt effectively in social contexts, thereby receiving more positive social feedback and reducing internalizing difficulties over time.

### 4.4 Chain-mediated effects of quality of sports friendships and social-emotional competence between physical activity and internalizing problems

This study demonstrates that sports friendship quality and SEC jointly serve as chain mediators between PA and internalizing problems, supporting Hypothesis 4. The quality of sports friendships was found to positively predict SEC development in adolescents with ASD. This result aligns with Arató et al. (2022), who reported that high-quality friendships can buffer negative emotions and promote adaptive emotional regulation and impulse control, thereby facilitating SEC. Specifically, adolescents with supportive peer relationships gain more social opportunities to practice emotional and social skills through interaction (Jesús Comellas Carbó, 2013). Thus, strong peer relationships contribute meaningfully to emotional recognition and social functioning, playing a critical role in the development of SEC.

Participation in structured, peer-based PA not only increases opportunities for companionship and higher-quality friendships, but also supports the development of SEC through shared goals and interaction (Kuo et al., 2013). Neurobiologically, PA helps redirect attention from internal states to external cues via multisensory input and motor coordination, thereby enhancing communication (Chen et al., 2018; Han et al., 2018). Activities such as horseback riding, martial arts, and swimming have been shown to significantly improve SEC in adolescents with ASD (Bremer et al., 2016). Through these sport-based friendships, adolescents develop emotional regulation, empathy, and perspective-taking, highlighting the role of PA as a meaningful pathway to strengthening SEC and mitigating internalizing problems.

## 5 Research limitations and future perspectives

This study has several limitations related to its measurement approach. First, the use of a cross-sectional design means that the relationships among variables can only be interpreted as correlational, preventing any inference about causal pathways or temporal sequence. Second, although the SDQ used in this study has a strong track record in assessing internalizing problems, its emotional symptoms subscale covers only anxiety and depression, which may limit its ability to comprehensively capture the full range of internalizing symptoms. As such, the internalizing problem scores should be interpreted with caution. Third, while adolescents with ASD may possess basic reading skills, some may not have fully understood the questionnaire items, potentially introducing bias into self-reported data. An additional limitation was the lack of information about the participants’ cognitive ability. Finally, the study did not incorporate important social-level factors such as interpersonal support and socioeconomic status, nor did it differentiate types of PA or examine participants’ attitudes and intrinsic motivation toward exercise. These unmeasured variables may also influence the relationship between PA and internalizing problems.

Future research should consider employing longitudinal designs to further clarify the causal relationship between PA and internalizing problems in adolescents with ASD. In addition, incorporating caregiver reports and including social-level variables such as social support, economic status, and family background would enhance the accuracy and external validity of the findings. Regarding the measurement of PA in adolescents with ASD, future studies should distinguish between different types of PA and combine self-reports with more objective tools, such as accelerometers, to reduce potential bias and improve data reliability.

## 6 Conclusion

This study explores the different pathways through which PA impacts internalizing problems in adolescents with ASD at the individual level, incorporating the variable of sports friendship quality into the analysis. The results show that the quality of sports friendships has a positive effect on internalizing problems in adolescents with ASD, providing new theoretical insights into the relationship between PA and internalizing problems in youth. Furthermore, both sports friendship quality and SEC serve as mediators in the relationship between PA and internalizing problems in adolescents with ASD, with both forming a serial mediation effect. This study highlights the significant value of these factors in adolescent psychopathology.

## Data Availability

The original contributions presented in the study are included in the article/supplementary material, further inquiries can be directed to the corresponding author.
